# The Old vs. the New

**Published:** 1875-10

**Authors:** 


### THE OLD VS. THE NEW.

BY OLD FOGY.

I am an old fogy, one that never catched at new remedies and modes simply because they were new. I bleed yet; would not practice medicine a day without the lancet. I purge yet in bilious attacks, and use the old "ten and ten." I blister, using the old home-made Spanish fly-plaster. I puke when necessary, using the old, much-abused tartar emetic. I use Peruvian bark as was wont by my preceptor thirty years ago. I use crude opium oftener than any of its preparations. I mustardise with a direct application of the mustard, made up with the white of an egg or mucilage of slippery elm or accacia, without putting muslin between plaster and surface. My success in treating disease fully vindicates my practice, and I am content. I have yet to give my first dose of hydro-chloral. Don't take worth a cent to the various new combinations and preparations recommended by the progressive doctors. But this article was not intended for a defense of these old time peculiarities which, like idiosyncrasy, will crop out, despite the great improvements that I notice daily in the medical journals. I love the good old way in which my fathers trod, and, being antiquated, am content with simple remedies, simple compounds, and simple appliances.

The object of this communication is simply to state that, in my experience, the old plan of making tinctures by maceration is the best. Preparations thus made are always reliable. I "macerate for fourteen days, then filter," and have never, when using tinctures thus prepared, been deceived in their therapeutic effect. I wish I could say the same of percolated tinctures. But candor compels me to say that, when forced to use them, I have as often been disappointed as otherwise in the effect sought to be produced. I like the old doctor's shop. We may not be scientific pharmaceutics, but in our rough, sledge-hammer style of preparing tinctures, we know what we are using, and can calculate, with some degree of certainty, upon the effect of the remedy prescribed. Then, Mr. Editor, give me the "old landmarks;" our fathers trod them safely before we were born; their bills of mortality were, in consequence, small, and we shall, at the risk of being forced to subscribe ourselves with the title which we assume, continue the same old beaten track.

